# Supplementary material for: Bacterial Diversity and Potential Functions in Response to Long-Term Nitrogen Fertilizer on the Semiarid Loess Plateau
Source: Microorganisms. 2022 Aug 5;10(8):1579. doi: 10.3390/microorganisms10081579 (PMC9412673; doi:10.3390/microorganisms10081579)
Supplement: Supplementary file 1 [file microorganisms-10-01579-s001.zip › microorganisms-1843958-supplementary.pdf]

Table S1. Detailed sequencing depth results of soil samples.

Sample	Repeats	clean_tags	valid_tags	OTU_counts	goods_coverage
N0	1	40484	32265	2988	0.970
N0	2	41515	33840	3149	0.968
N0	3	38389	31842	3026	0.969
N52.5	1	47904	36622	3228	0.969
N52.5	2	41739	34177	3085	0.969
N52.5	3	37392	31575	3024	0.969
N105	1	44946	36574	3267	0.968
N105	2	44299	33220	3178	0.968
N105	3	43082	35497	3178	0.969
N157.5	1	38378	30498	2826	0.972
N157.5	2	48571	37504	3218	0.969
N157.5	3	49879	37083	3242	0.969
N210	1	41990	32162	2902	0.970
N210	2	36481	30699	2932	0.971
N210	3	40539	30174	3055	0.968
N52.5c	1	38903	31966	3057	0.968
N52.5c	2	35004	31250	3062	0.968
N52.5c	3	47799	39145	3147	0.970
N105 c	1	36947	32891	3198	0.967
N105c	2	42309	30770	2701	0.972
N105c	3	46061	36258	3091	0.970
N157.5c	1	38528	29812	3042	0.968
N157.5c	2	46700	36005	3247	0.969
N157.5c	3	42297	34301	3089	0.968
N210c	1	38675	30698	3027	0.969
N210c	2	33244	28900	2989	0.969
N210c	3	42615	35431	3159	0.969

N0, 0 kg N ha^{-1} $year^{-1}$; N52.5, 52.5 kg N ha^{-1} $year^{-1}$; N105, 105 kg N ha^{-1} $year^{-1}$; N157.5, 157.5 kg N ha^{-1} $year^{-1}$; N210, 210 kg N ha^{-1} $year^{-1}$; N52.5c, 52.5 kg N ha^{-1} 2 year; N105c, 105 kg N ha^{-1} 2 year; N157.5c, 157.5 kg N ha^{-1} 2 year; N210c, 210 kg N ha^{-1} 2 year.

Table S2. Relative abundance of soil bacterial taxonomic composition at phyla level for all samples.

Treatment	Proteobacteria	Actinobacteria	Acidobacteria	Chloroflexi	Gemmatimonadetes	Bacteroidetes	Nitrospirae	Verrucomicrobia	Planctomycetes
N0	23.26±0.53c	20.83±0.34a	21.14±0.49a	13.53±0.41a	8.40±0.12c	3.67±0.33b	1.68±0.04e	1.74±0.04a	1.42±0.05a
N52.5	23.15±0.54c	20.76±0.23a	19.30±0.35bc	13.97±0.40a	9.12±0.31b	4.52±0.45ab	1.80±0.04de	1.69±0.07a	1.22±0.10ab
N105	22.64±0.51c	19.57±0.30bc	21.34±0.44a	12.94±0.13ab	9.21±0.15b	4.33±0.43ab	2.22±0.03a	1.86±0.04a	1.31±0.06a
N157.5	27.08±0.36b	19.96±0.35abc	16.12±0.04d	11.27±0.70c	10.04±0.24a	4.77±0.24ab	1.85±0.04cd	1.18±0.04b	1.07±0.04bc
N210	28.84±0.53a	19.27±0.35c	17.68±0.53cd	11.87±0.22bc	9.13±0.24b	5.37±0.27a	1.87±0.03cd	0.86±0.05c	0.85±0.07c
N52.5[c]	23.33±0.74c	20.49±0.33ab	20.28±0.58ab	13.60±0.59a	8.42±0.10c	3.94±0.37b	1.74±0.04de	1.70±0.05a	1.02±0.07bc
N105[c]	23.48±0.59c	20.17±0.41abc	19.32±0.67bc	13.44±0.37a	9.41±0.26b	4.09±0.33ab	1.86±0.05cd	1.66±0.16a	0.99±0.09c
N157.5[c]	25.38±0.79b	19.76±0.50abc	19.48±0.83b	13.11±0.80ab	9.24±0.07b	4.33±0.42ab	1.96±0.07bc	1.70±0.09a	1.02±0.04bc
N210[c]	26.47±0.56b	19.38±0.22bc	21.54±0.44a	13.10±0.26ab	9.38±0.10b	4.73±0.60ab	2.03±0.04b	1.84±0.07a	1.34±0.04a
ANOVA *P*-value	＜0.000	0.033	＜0.000	0.017	＜0.000	0.185	＜0.000	＜0.000	＜0.000

N0, 0 kg N ha^{-1} $year^{-1}$; N52.5, 52.5 kg N ha^{-1} $year^{-1}$; N105, 105 kg N ha^{-1} $year^{-1}$; N157.5, 157.5 kg N ha^{-1} $year^{-1}$; N210, 210 kg N ha^{-1} $year^{-1}$; N52.5c, 52.5 kg N ha^{-1} 2 year; N105c, 105 kg N ha^{-1} 2 year; N157.5c, 157.5 kg N ha^{-1} 2 year; N210c, 210 kg N ha^{-1} 2 year. Data (means ± SD, n = 3) labeled with different letters are significant at $p < 0.05$.

Table S3. Redundancy analysis results of soil physiochemical properties affecting the distribution of dominant phyla and clustering of soil samples.

Soil property	Explains (%)	Contribution (%)	*F*	*P*
NO_3-N	49.8	80.7	24.8	0.002
AP	3.6	5.8	1.8	0.136
Moisture	2.8	4.5	1.5	0.230
pH	1.4	2.3	0.7	0.574
TN	1.7	2.7	0.9	0.480
TP	1.3	2	0.6	0.642
NH_4-N	1.2	2	0.6	0.618

AP, available phosphorus; TN, total N; TP, total phosphorus.

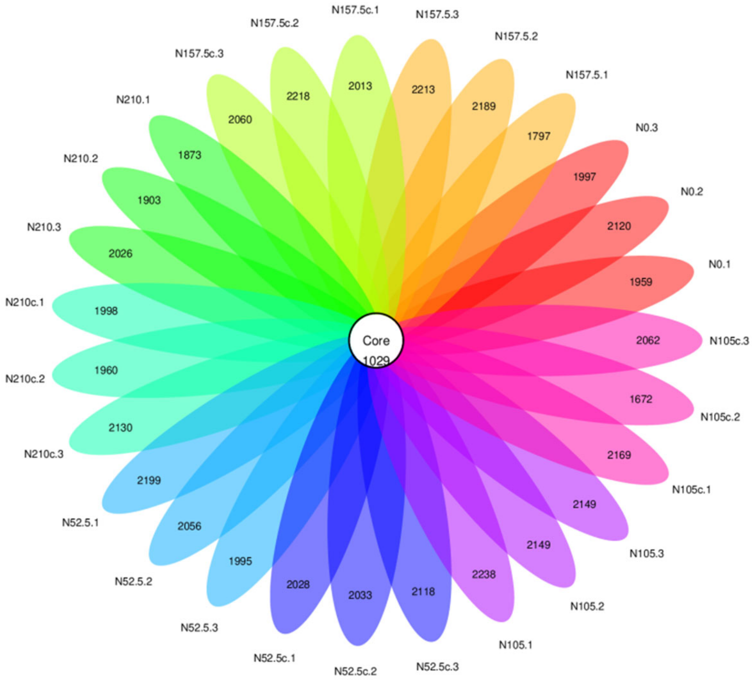

Figure S1. Operational taxonomic unit Venn analysis of unique and shared bacteria 16S rRNA as affected by N fertilization. N0, 0 kg N ha^{-1} $year^{-1}$; N52.5, 52.5 kg N ha^{-1} $year^{-1}$; N105, 105 kg N ha^{-1} $year^{-1}$; N157.5, 157.5 kg N ha^{-1} $year^{-1}$; N210, 210 kg N ha^{-1} $year^{-1}$; N52.5c, 52.5 kg N ha^{-1} 2 year; N105c, 105 kg N ha^{-1} 2 year; N157.5c, 157.5 kg N ha^{-1} 2 year; N210c, 210 kg N ha^{-1} 2 year.

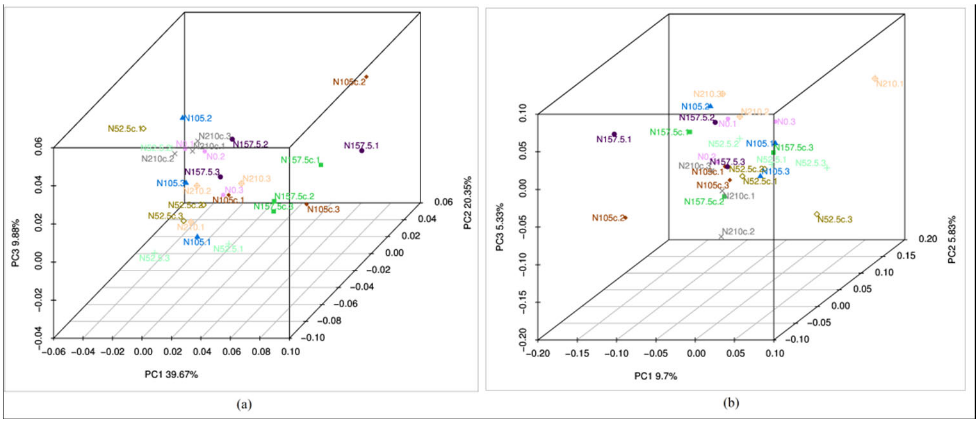

Figure S2. Summary of principal coordinate analysis of soil bacterial composition based on (a) the weighted UniFrac and (b) unweighted UniFrac. N0, 0 kg N ha^{-1} year^{-1}; N52.5, 52.5 kg N ha^{-1} year^{-1}; N105, 105 kg N ha^{-1} year^{-1}; N157.5, 157.5 kg N ha^{-1} year^{-1}; N210, 210 kg N ha^{-1} year^{-1}; N52.5c, 52.5 kg N ha^{-1} 2 year; N105c, 105 kg N ha^{-1} 2 year; N157.5c, 157.5 kg N ha^{-1} 2 year; N210c, 210 kg N ha^{-1} 2 year.

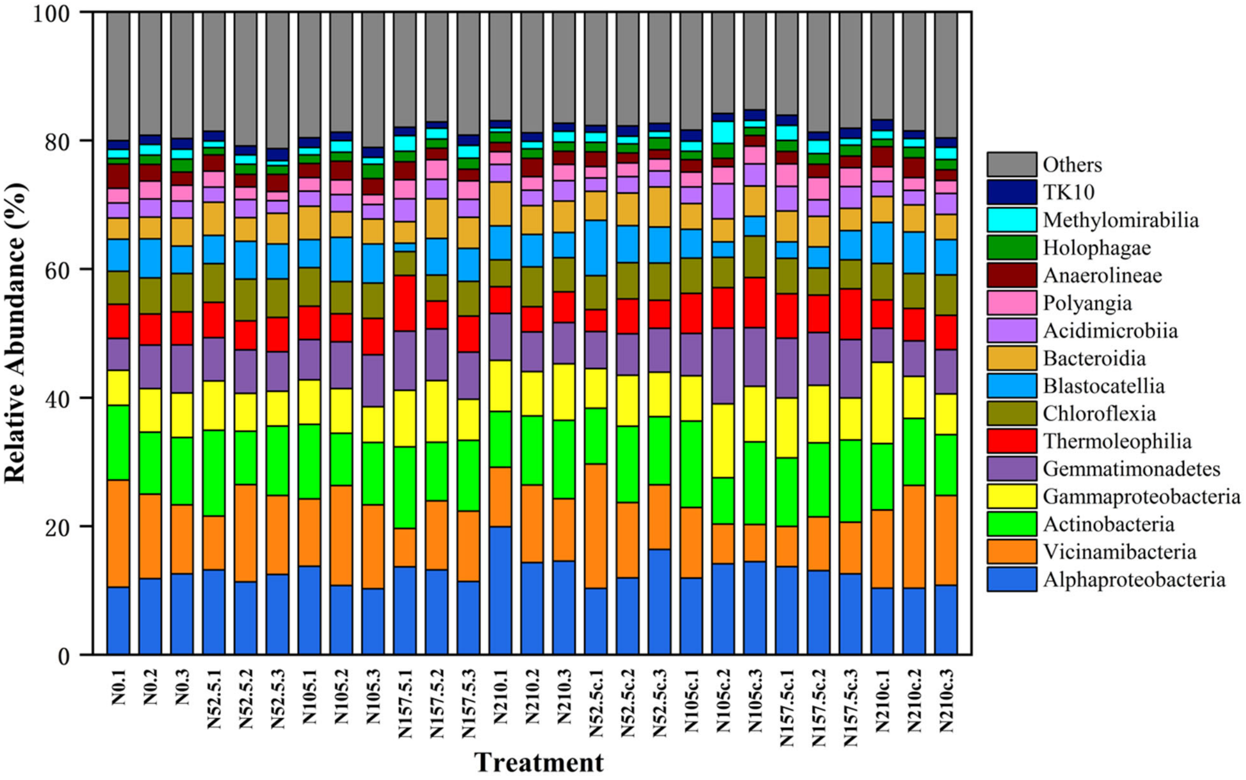

Figure S3. Relative abundance of top 15 soil bacterial class for all samples. N0, 0 kg N ha^{-1} year^{-1}; N52.5, 52.5 kg N ha^{-1} year^{-1}; N105, 105 kg N ha^{-1} year^{-1}; N157.5, 157.5 kg N ha^{-1} year^{-1}; N210, 210 kg N ha^{-1} year^{-1}; N52.5c, 52.5 kg N ha^{-1} 2 year; N105c, 105 kg N ha^{-1} 2 year; N157.5c, 157.5 kg N ha^{-1} 2 year; N210c, 210 kg N ha^{-1} 2 year. Treatments labeled with .1, .2, and .3 represent 3 repetitions.

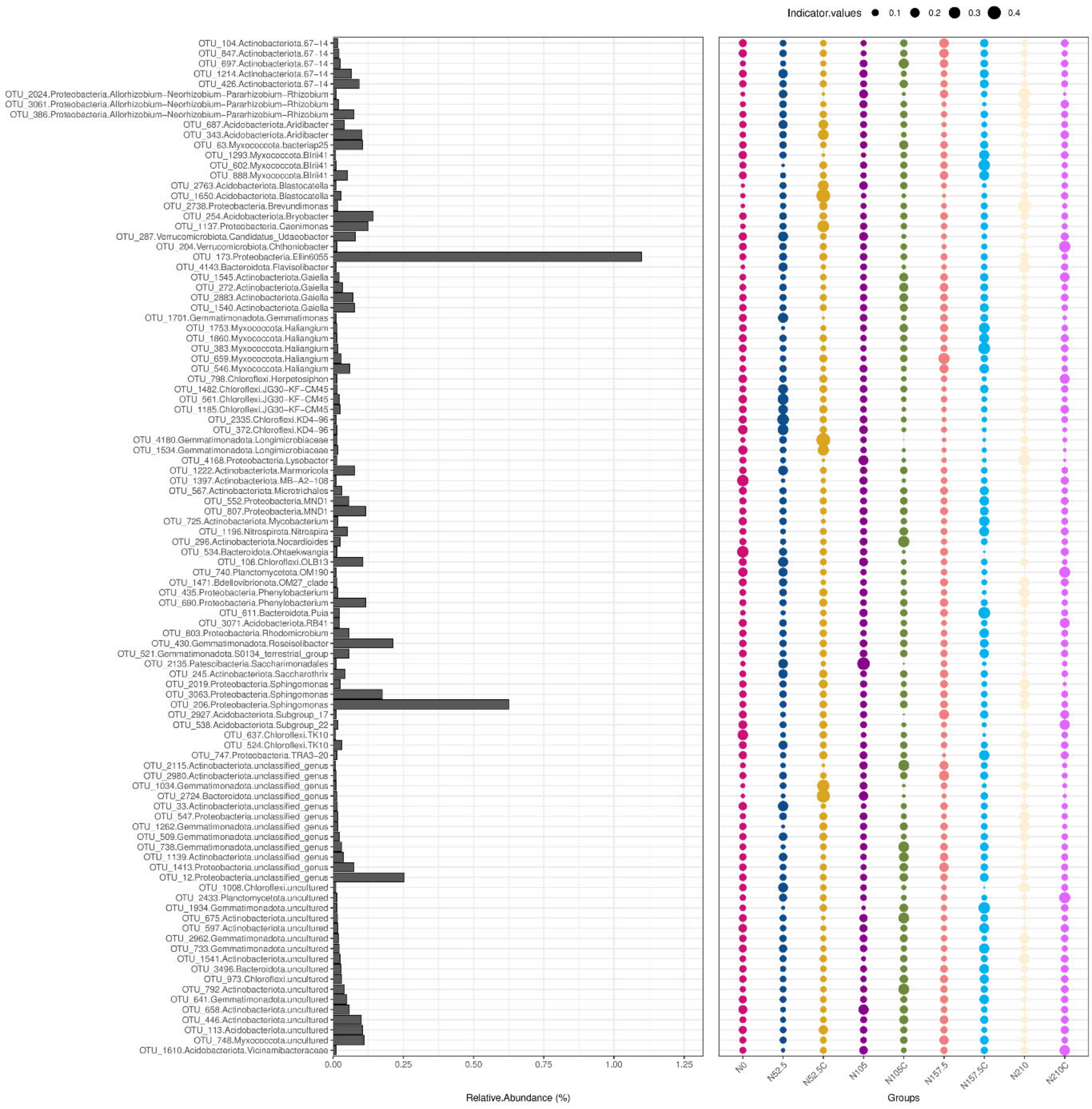

Figure S4. Indicator species of soil bacterial genus for all samples as affected by N fertilization. The first column shown in the figure represents OTU, phyla level, and genus level information; the histogram indicates the relative abundance of each OTU; the abscissa of the bubble chart indicates the sample grouping (treatment), and the size of each bubble represents the size of the indicator value in each group.
